# Surface Energy Driven Cubic-to-Hexagonal Grain Growth of Ge_2_Sb_2_Te_5_ Thin Film

**DOI:** 10.1038/s41598-017-06426-2

**Published:** 2017-07-19

**Authors:** Yonghui Zheng, Yan Cheng, Rong Huang, Ruijuan Qi, Feng Rao, Keyuan Ding, Weijun Yin, Sannian Song, Weili Liu, Zhitang Song, Songlin Feng

**Affiliations:** 1 State Key Laboratory of Functional Materials for Informatics, Shanghai Institute of Micro-system and Information Technology, Chinese Academy of Sciences, Shanghai, 200050 China; 2 University of the Chinese Academy of Sciences, Beijing, 100049 China; 3 Key Laboratory of Polar Materials and Devices Ministry of Education, East China Normal University, Shanghai, 200062 China

## Abstract

Phase change memory (PCM) is a promising nonvolatile memory to reform current commercial computing system. Inhibiting face-centered cubic (f-) to hexagonal (h-) phase transition of Ge_2_Sb_2_Te_5_ (GST) thin film is essential for realizing high-density, high-speed, and low-power PCM. Although the atomic configurations of f- and h-lattices of GST alloy and the transition mechanisms have been extensively studied, the real transition process should be more complex than previous explanations, e.g. vacancy-ordering model for f-to-h transition. In this study, dynamic crystallization procedure of GST thin film was directly characterized by *in situ* heating transmission electron microscopy. We reveal that the equilibrium to h-phase is more like an abnormal grain growth process driven by surface energy anisotropy. More specifically, [0001]-oriented h-grains with the lowest surface energy grow much faster by consuming surrounding small grains, no matter what the crystallographic reconfigurations would be on the frontier grain-growth boundaries. We argue the widely accepted vacancy-ordering mechanism may not be indispensable for the large-scale f-to-h grain growth procedure. The real-time observations in this work contribute to a more comprehensive understanding of the crystallization behavior of GST thin film and can be essential for guiding its optimization to achieve high-performance PCM applications.

## Introduction

As a promising candidate for storage-class memory^1^ to mitigate the performance gap between dynamic random access memory (DRAM) and NAND Flash memory^2^, phase change memory (PCM) bears excellent properties including sub 10 ns switching speed^3, 4^, scalability to sub 10 nm dimension^5, 6^, more than 10^11^ cyclability^6^, and up to 220 °C 10-year data retention ability^7^. In commercialized PCM devices, the reversible transitions between amorphous (a-) and crystalline (c-) phases of Ge_2_Sb_2_Te_5_ (GST) material are utilized to store “0” and “1” data states. The RESET operation refers to an amorphization procedure which melts the c-phase and subsequently quenches it to a-phase by applying a short intense electrical pulse on PCM device. Conversely, a longer pulse of lower intensity for SET operation can heat the a-phase to a temperature between crystallization and melting points to obtain c-structure.

The a-GST material firstly crystallizes into a metastable face-centered-cubic (f-) lattice structure at ~150 °C^8^, and subsequently transforms into equilibrium hexagonal (h-) structure at 300~350 °C^9^. In f-GST lattice, Te atoms occupy well-defined anionic sites, while Ge/Sb atoms and vacancies randomly occupy the cationic sites^10^; that is, the atomic stacking sequence along [111] direction of f-lattice ([0001] direction of h-lattice) is –(Te–Ge/Sb/Vacancy)_n_–^10–14^. While in h-GST, along the [0001] direction, the stacking feature of nine-layered building block can be described as –(Te–Ge/Sb–Te–Ge/Sb–Te–Te–Ge/Sb–Te–Ge/Sb)_n_–^14^. Avoiding f-to-h phase transition of GST alloy has always been a key issue in PCM technology field, since in a real PCM device, the h-GST phase is deemed to be a black sheep incurring larger RESET current, slower SET speed, severe composition drift, bigger volume shrinkage upon re-crystallization, and large void formation after high temperature (~400 °C) back-end of line process^15, 16^.

Recently, an intermediate state of f-GST, namely vacancy-ordering cubic (VOC) phase, was theoretically^17^ and experimentally^11, 12^ demonstrated to play a key role during the f-to-h transition. Once 100% vacancy-aggregation into {111} planes achieves and forms non-atomic layers in f-GST resembling the Van der Waals interaction gaps in h-GST, the system energy is as small as the equilibrium h-phase^17^. In view of the similar configuration and minor energy discrepancy between the 100% VOC- and h-GST lattices, a non-diffusion controlled slide of the building blocks was proposed to understand the f-to-h transformation^18^. Analogous research achievement stated that a shearing martensitic transformation from {200} planes of f-GST to {$10\bar{1}3$} planes of h-GST should be energetically favorable during f-to-h transition^19, 20^. In addition, the discovery of twin crystals consisted by one f- and one h-grain led to an “epitaxial growth model” to interpret the structure evolution manner as f-GST approaching the h-phase^21, 22^. One can find that previous studies inclined to utilize transformation between similarly structured f- and h-lattices (often from specific crystal orientations under static observation) to conjecture the dynamic atomic rearrangements for the whole f-to-h transition, which would inevitably be neither comprehensive nor precise enough.

In this report, *in situ* heating transmission electron microscopy (TEM) was utilized to characterize the dynamic crystallization procedure of GST thin films. We note previous literature^23^ mainly concentrated on the phase transformation procedure and electronic structure of GST films upon *in situ* annealing, while no vacancy ordering process or h-grain growth mechanism was discussed. Here, we reveal that the rapid growth behavior of h-grains for GST thin film resembles an “abnormal” type^24, 25^, which is driven by surface energy anisotropy. In contrast to the “normal” case in which grains get larger in a uniform manner, the abnormal growth of h-grains can be characterized by a subset of h-grains (mainly [0001]-oriented) growing bigger at a high rate and at the expense of their multifarious neighboring (small) grains. Such swift expansion of the big h-grain was usually named as the “growth-dominated crystallization” for f-to-h transition of GST^16, 21^, no matter what crystallographic configurations the small grains would have. We also speculate that the vacancy-ordering process into {111} planes^11, 12, 17^ may or may not occur in every f-grain, or to say, it is not an indispensable way for f-grains evolving into h-ones especially in the growth period. The adjustments by sliding the building blocks between similar f- and h-atomic configurations^17, 19, 21^ would be more likely to involve the incubation of h-seeds from the f-matrix. The present scenarios may offer a more comprehensive perspective to understand the phase transition physics of this key material, and be essential for optimizing GST-based commercialized phase change materials to boost the performances of high density PCM device.

## Results

### *In situ* heating crystallization of Ge_2_Sb_2_Te_5_ thin film

**Figure 1:**
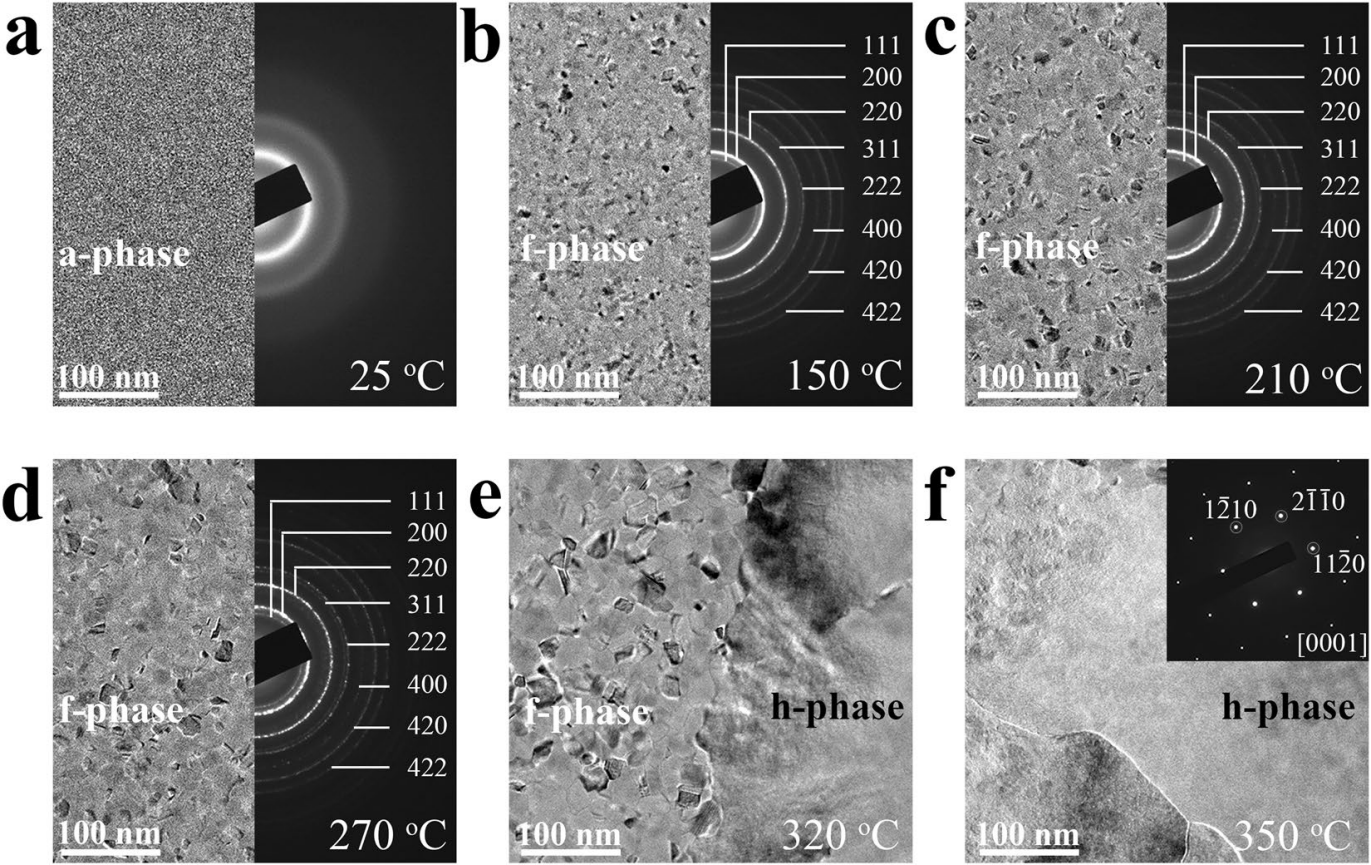
*In situ* heating crystallization of as deposited Ge_2_Sb_2_Te_5_ thin film. (**a**–**d**) bright-field transmission electron microscopy images and the corresponding selected area electron diffraction (SAED) patterns at different temperatures (25 °C, 150 °C, 210 °C, and 270 °C, respectively). (**e**) The coexistence of a large hexagonal (h-) grain and small grains on the other side of the grain boundary at 320 °C. (**f**) The final morphology and its corresponding SAED pattern of the large single-crystal type h-grain with strong [0001] texture.

Figure 1 exhibits *in situ* heating crystallization process of GST thin film at different temperatures in TEM. The as-deposited GST thin film (Fig. 1a) shows typical a-phase at room temperature. It crystallizes into f-phase with uniformly distributed (randomly oriented) nano-crystals (<~15 nm in average grain size) at 150 °C (Fig. 1b) with calculated lattice parameter of *a* = 6.01 Å^16, 26^. When temperature increases to 210 °C (Fig. 1c) and 270 °C (Fig. 1d), f-grains continuously grow larger as the average grain size reaching ~20 and ~30 nm, respectively. The corresponding selected area electron diffraction (SAED) rings in Fig. 1c,d are of a little discontinuity as compared to that in Fig. 1b, denoting the gradual enlargements of the f-grains at higher temperatures. The transient moment at 320 °C (Fig. 1e) shows that GST film has small grains (mixture of evenly distributed small f- and h-grains as also proved in Supplementary Fig. 1) bordering a large h-grain. The dominant h-grain quickly swallows (with ~6.7 nm/s growing speed) adjacent small grains like flood and grows into a larger one, as clearly recorded in Supplementary Movie 1 (with the observation area ~2 × 2 μm^2^). The large h-grain with μm size, as illustrated from the SAED pattern in Fig. 1f, is of single-crystal type showing strong [0001]-oriented texture. Since we also found tiny h-grains can be incubated from f-matrix at pretty low temperature (~210 °C) (Supplementary Fig. 2), it is reasonable to deduce that, under the circumstance of favorable energy level and similar atomic configuration^17–22^, some of them may act as seeds for quickly growing up into the dominant [0001]-oriented h-grains.

### Unnecessity of vacancy-ordering mechanism at fast growth stage of hexagonal grain

**Figure 2:**
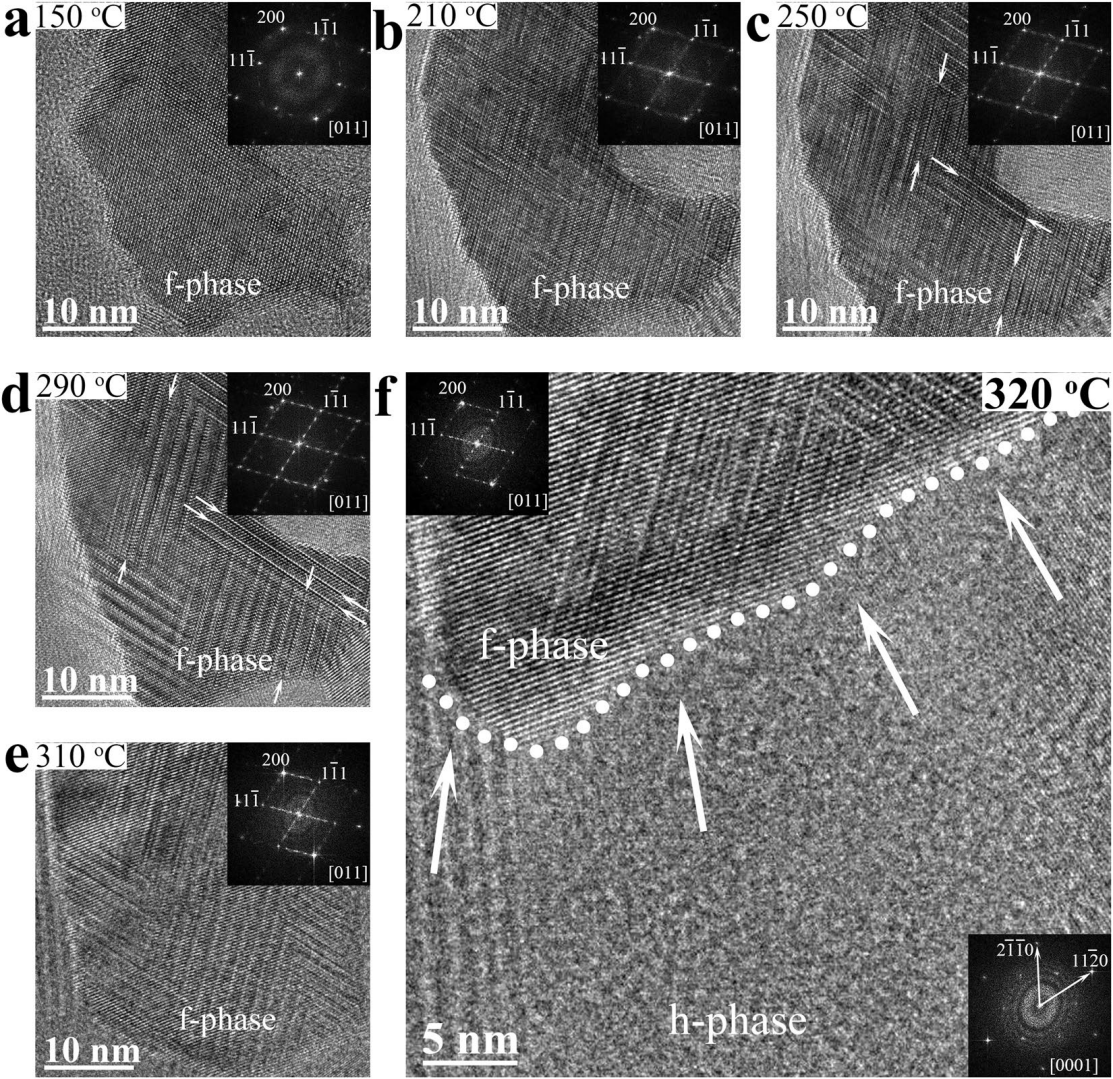
*In situ* heating vacancy-ordering process occurred in a [011]-oriented face-centered cubic grain. (**a**–**e**) High resolution transmission electron microscopy (HRTEM) snapshots and corresponding fast-Fourier transform patterns at different heating temperatures, showing the gradual ordering of vacancies in the face-centered cubic (f-) grain. (**f**) Enlarged HRTEM image shows a transient moment on the grain boundary between such f-grain and a [0001]-oriented large hexagonal (h-) grain. Although the [011]-oriented f-grain and the [0001]-oriented h-grain are not parallel, the f-grain is going to be consumed by its large neighbor in subsequent heating process.

In Fig. 2, we reveal the *in situ* heating vacancy aggregation on {111} planes in a [011]-oriented f-grain, and finally being swallowed by an adjacent large h-grain. At 150 °C (Fig. 2a), the observed grain shows typical f-lattice structure with randomly distributed vacancies. When temperature reaches 210 °C (Fig. 2b), some intersecting (parquet-like) defect layers^26^ paralleling to {111} planes emerge, causing weak scattered streaks in the inserted corresponding fast-Fourier transform (FFT) pattern. These defect layers should be ascribed to the ordering of randomly distributed vacancies in f-lattice^27, 28^. In the case of 250~290 °C (Fig. 2c,d), further vacancy ordering leads to gradually clearer and longer defect layers (marked by arrows) in the f-grain. The brighter super-lattice reflection spots in corresponding FFT pattern represent a long-period ordered structure separated by the defect layers. At this period, the f-grain corresponds to an incomplete vacancy-ordering status, and there are still plenty of (Ge/Sb) atoms resided in the van der Waals-like gaps^11^. At 310 °C (Fig. 2e), the f-grain becomes a little larger, while its high resolution transmission electron microscopy (HRTEM) and FFT images become a bit blurred, which may be originated from a slight grain rotation, resulting in the deviation of [011] zone axis from the incident direction of the electron beam.

As heating temperature increases to 320 °C (Fig. 2f), the [011]-oriented f-grain is about to be eaten by an adjacent big [0001]-oriented h-grain. It is worth pointing out that the non-diffusion controlled slide of building blocks between similar f- and h- atomic configurations requires parallelism between 〈111〉_f_ and 〈0001〉_h_ directions^17, 18^. Nevertheless in current case the [011]-oriented f-grain deviates ~35.3° (inter-axial angle) with 〈111〉_f_ direction^29^ (standing for 〈0001〉_h_ direction). Obviously, the non-diffusion controlled slide model is invalid here for the largely misaligned structure rearrangement.

### Transient growth state on hexagonal grain boundary

**Figure 3:**
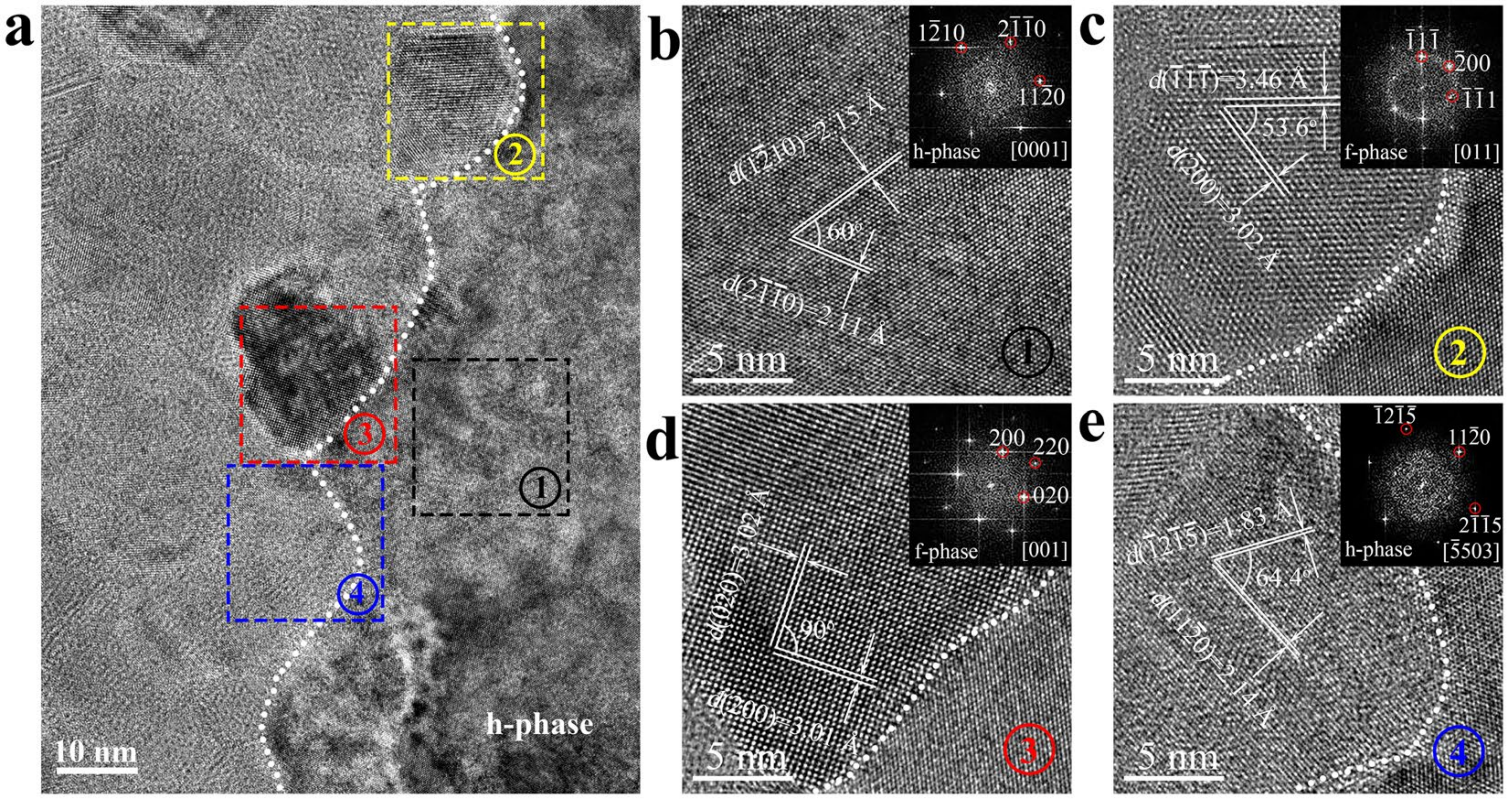
Complex transient growth state on the hexagonal grain boundary. (**a**) High resolution transmission electron microscopy (HRTEM) snapshot of the face-centered cubic (f-) to hexagonal (h-) transient moment exhibits a large [0001]-oriented h-grain on the right side of the white dotted grain boundary and randomly oriented small crystal grains on the left side. (**b**–**e**) Magnified HRTEM images and the corresponding fast-Fourier transform patterns of the framed regions in (**a**) denoted as ①, ②, ③, and ④ respectively. The [011]-oriented f-grain in region ② without obvious vacancy-ordered layers, the [001]-oriented f-grain in region ③, and the $[\bar{5}503]$-oriented h-grain in region ④ all will be transformed into (consumed by) the [0001]-oriented large h-grain.

On the boundary of a dominant [0001]-oriented h-grain (see Fig. 3a and zooming image of region ① in Fig. 3b), there reside some small grains corresponding to selected regions ②~④ (see Fig. 3a and zooming images in Fig. 3c to e respectively), which was captured at room temperature from an annealed sample heated up to 320 °C in TEM. Through indexing, the small grains in regions ②~④ are [011]-oriented f-grain, [001]-oriented f-grain, and $[\bar{5}503]$-oriented h-grain, respectively. In contrast to the vacancy-ordered f-grain (in Fig. 2f), f-grain in region ② has not undergone obvious vacancy-ordering process, but it still cannot escape being annexed by the dominant h-grain as heating continues. The complexities are also produced by f-grain in region ③ and h-grain in region ④. For the [001]-oriented f-grain in region ③ which is unparallel to the 〈0001〉_h_ direction, whether or not the vacancy-ordered layers exist, it could not be transformed into h-phase via a simple building-block sliding procedure. The $[\bar{5}503]$-oriented h-grain in region ④ also misaligns with the dominant [0001]-oriented h-grain, and the calculated inter-axial angle between $\langle \bar{5}503\rangle$ and 〈0001〉 orientations is about 35 6° (ref. 29). In addition, a well-resolved video in Supplementary Movie 2 (with the observation area ~20 × 20 nm^2^) shows the quick expansion (in a few seconds) of a [0001]-oriented h-grain from the upper part by consuming a subjacent grain (without any grain-rotation). The consumed grain obviously does not belong to the vacancy-ordered type. These results further prove that vacancy-ordering into {111} planes of the f-grain may or may not happen especially at the stage of fast h-grain growth. We thus believe the VOC-to-h rearranging model is not enough to describe the f-to-h transition image, and it would be more or less only related to the stochastic (energetically favorable) h-nucleation from f-matrix.

## Discussion

**Figure 4:**
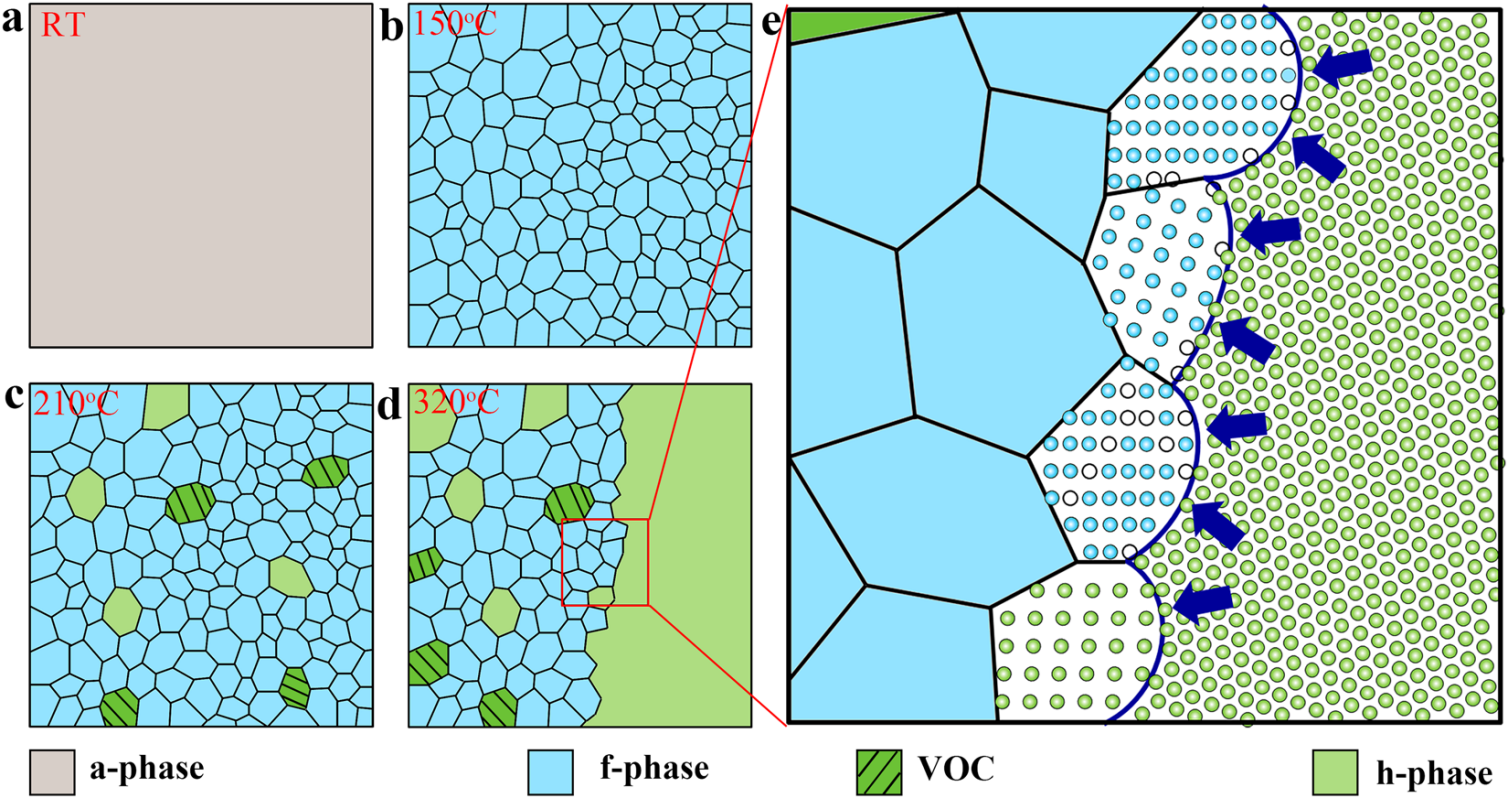
Schematic drawings of the crystallization procedure of Ge_2_Sb_2_Te_5_ thin film. (**a**) Amorphous phase at room temperature (RT). (**b**) Face-centered cubic (f-) phase at ~150 °C. (**c**) Small vacancy-ordered f- and incubated hexagonal (h-) grains emerge at above ~210 °C. (**d**) Specific transient moment shows the co-existence of a dominant [0001]-oriented h-grain and multi-oriented small f- or h-grains at ~320 °C. (**e**) Enlarged sketch illustrates the complex crystallography on the boundary of the dominant [0001]-oriented h-grain shown in (**d**). The arrows specify the grain growth directions, and the open circles represent vacant atom sites in f-grain.

Given this, we summarize the crystallization process of GST thin film in Fig. 4. The a-GST (Fig. 4a) firstly crystallizes into small f-grains with randomly distributed vacancies at ~150 °C (Fig. 4b). Then, some randomly oriented VOC-grains and small h-grains are formed from the f-matrix at ~210 °C (Fig. 4c). By further heating up to ~320 °C (Fig. 4d), a dominant (large) [0001]-oriented h-grain appears and grows up quickly to accomplish the f-to-h transformation. The enlarged sketch (Fig. 4e) illustrates the transient growth state on the boundary of the dominant h-grain. Since the concentration of adatoms on the grain boundary is a function of its curvature^30^, usually atoms from the convex side tend to increase their coordination number via migrating to the concave side so as to lower down the system free energy. This is also valid here when low-coordinated atoms in f-GST lattice with large portion of vacancies confront the compact h-lattice. They diffuse to the boundary and reconstruct into the high-coordinated configuration. The arrows marked in Fig. 4e denote the concave-to-convex expansion (growth) directions for the dominant h-grain.

In theory, the grain growth rate *V* can be expressed as:

1 $$V=M\cdot F,$$

where *M* is the average grain boundary mobility and *F* is the combined driving force^25, 31^. The latter can be qualitatively divided into three categories: surface energy, grain boundary energy, and free energy from f-to-h phase transformation.

**Figure 5:**
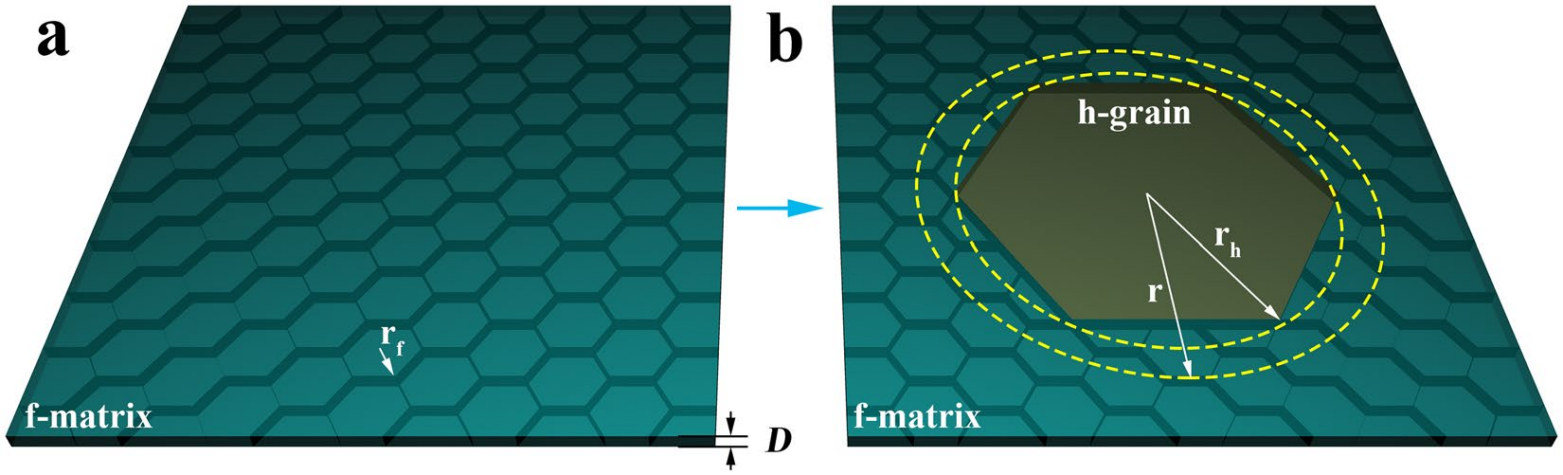
Schematic drawings showing simplified geometrical characteristics of abnormal grain growth. (**a**) The initial face-centered cubic (f-) matrix with uniform f-grain size *r*_*f*_ and film thickness *D*. (**b**) Abnormal hexagonal (h-) grain growth moment provides the starting h-grain size of radius *r*_*h*_ embedded in f-matrix and a subsequent size of radius *r*.

To model the abnormal h-grain growth in f-GST thin film of thickness *D* (Fig. 5), we examine the case that an initial h-GST grain of radius *r*_*h*_ is incubated from the f-GST matrix with uniform f-grain radius *r*_*f*_ (Fig. 5a), and grows into a bigger one of radius *r* (Fig. 5b). The starting h-grain can be characterized by a surface energy per unit area Γ_*s*_,_*h*_ and an interphase boundary energy between the h- and f-phases per unit area *Γ*_*f*/*h*_. The f-grains are characterized by an average grain boundary energy per unit area ${{\rm{\Gamma }}}_{gb,f}^{\ast }$ and average surface energy per unit area ${{\rm{\Gamma }}}_{s,f}^{\ast }$. Note that ${{\rm{\Gamma }}}_{s,h}$, Γ_*f*/*h*_, ${{\rm{\Gamma }}}_{s,f}^{\ast }$, and ${{\rm{\Gamma }}}_{gb,f}^{\ast }$ all have positive values and obviously ${{\rm{\Gamma }}}_{s,f}^{\ast } > {{\rm{\Gamma }}}_{s,h}$.

For the h-grain of radius *r*_*h*_ to annex its surrounding f-grains contained in an area of $\pi {r}^{2}-\pi {r}_{h}^{2}$, the energy per unit volume of this region before the subsequent grain growth is:

2 $$\begin{array}{ccc}{E}_{B} & = & \{[2\pi {r}_{h}^{2}{{\rm{\Gamma }}}_{s,h}+2(\pi {r}^{2}-\pi {r}_{h}^{2}){{\rm{\Gamma }}}_{s,f}^{\ast }]\\  &  & +\,2\pi {r}_{h}D{{\rm{\Gamma }}}_{f/h}+(\pi {r}^{2}-\pi {r}_{h}^{2})DN{A}_{gb}{{\rm{\Gamma }}}_{gb,f}^{\ast }\\  &  & +\,[{\rm{\Delta }}{G}_{h}\pi {r}_{h}^{2}D+{\rm{\Delta }}{G}_{f}(\pi {r}^{2}-\pi {r}_{h}^{2})D]\}/\pi {r}^{2}D,\end{array}$$

where $\,N=1/(\pi {r}_{f}^{2}D)$ is the total number of f-grains per unit volume, ${A}_{gb}=\pi {r}_{f}D$ is the grain boundary area associated with an average f-grain, and Δ*G*_*h*_ and Δ*G*_*f*_ are the free energies of formation per unit volume of h- and f-GST respectively. Apparently, Δ*G*_*h*_ and Δ*G*_*f*_ are both negative, and Δ*G*_*f*_  > Δ*G*_*h*_. After transformation, the fast growth of the dominant h-grain achieves significant reduction of the total energy, and the energy at this time of this region can be described as:

3 $${E}_{A}=(2\pi {r}^{2}{{\rm{\Gamma }}}_{s,h}+2\pi rD{{\rm{\Gamma }}}_{f/h}+{\rm{\Delta }}{G}_{h}\pi {r}^{2}D)/\pi {r}^{2}D.$$

The driving force for the transformation from the higher energy state *E*_*B*_ to the lower one *E*_*A*_ can therefore be expressed as:

4 $$\begin{array}{ccc}F={E}_{B}-{E}_{A} & = & \frac{2}{D}(1-\frac{{r}_{h}^{2}}{{r}^{2}})({{\rm{\Gamma }}}_{s,f}^{\ast }-{{\rm{\Gamma }}}_{s,h})\\  &  & +\,(1-\frac{{r}_{h}}{r})\,[-\frac{2{{\rm{\Gamma }}}_{f/h}}{r}+\,(1+\frac{{r}_{h}}{r})N{A}_{gb}{{\rm{\Gamma }}}_{gb,f}^{\ast }]\\  &  & +\,({\rm{\Delta }}{G}_{f}-{\rm{\Delta }}{G}_{h})(1-\frac{{r}_{h}^{2}}{{r}^{2}}).\end{array}$$

In equation (4), the first, second, and the third terms represent the contributions from surface energy, grain-boundary energy, and free energy change from f-to-h transformation respectively.

At the initial heating stage of transforming a f-grain into a small h-grain, when *r*_*h*_ ≈ 0 and *r* ≈ *r*_*f*_, the driving force can be roughly described as:

5 $$F=2({{\rm{\Gamma }}}_{s,f}^{\ast }-{{\rm{\Gamma }}}_{s,h})/D+({{\rm{\Gamma }}}_{gb,f}^{\ast }-2{{\rm{\Gamma }}}_{f/h})/{r}_{f}+({\rm{\Delta }}{G}_{f}-{\rm{\Delta }}{G}_{h}).$$

From equation (5), we notice that the surface energy anisotropy and Gibbs free energy change upon f-to-h phase transition represent the positive momentums. As for the interphase boundary energy, no doubt it would act as a barrier to h-grain growth at the initial stage and represent the negative momentum, especially when the average f-grain size *r*_*f*_ is pretty small. It has been demonstrated that the modified GST materials, eg., after Ge^15, 32^, C^16^, or N^32, 33^ doping, all have diminished f-grains. According to equation (5), when the grain size is pretty small, we can roughly regard the surface energy anisotropy as a constant parameter, and a smaller *r*_*f*_ of f-grain in modified GST films would make the interphase boundary energy become dominant as transforming to h-grain. Not surprisingly, the transformation to h-phase thus can be postponed to higher temperature. In addition, the formation of segregated Ge, C, or GeN_x_ phases on the grain boundaries can significantly inhibit the h-grain boundary migration (*M* being lowered), leading to the slow grain growth rate of h-phase.

On the other hand, for the fast growth period of the dominant h-grain, consuming surrounding small f-grains, the driving force, by assuming *r* ≈ *r*_*h*_ + *r*_*f*_ and *r* ≈ *r*_*h*_ (>2 μm) ≫ ${r}_{f}$ (~20 nm), then can be rewritten into:

6 $$F=4{r}_{f}({{\rm{\Gamma }}}_{s,f}^{\ast }-{{\rm{\Gamma }}}_{s,h})/rD+(-2{r}_{f}{{\rm{\Gamma }}}_{f/h})/{r}^{2}+2{{\rm{\Gamma }}}_{gb,f}^{\ast }/r+2{r}_{f}({\rm{\Delta }}{G}_{f}-{\rm{\Delta }}{G}_{h})/r$$

According to equation (6), one can qualitatively conclude that, for the ultra-thin GST film (*D* being quite small), the contribution of surface energy anisotropy (the first item) becomes extremely dominant as the average grain size is greater than the film thickness^31^. In fact, at higher temperature with larger *r*_*f*_ grain size, the contribution of surface anisotropy would be more dominant, which in turn increases driving force and prompts the h-grain growth. In this case, grains with orientation of low surface energy will preferentially grow faster than those with other orientations. The abnormal grain growth thus prevails until the orientation-favored grains impinge on grains with similar surface energy. Eventually, most of the grains surviving abnormal grain growth would be orientated with the minimum surface energy. In c-materials, the minimum surface energy planes usually are the closest-packed atomic ones^31^, eg., (0001) plane of h-GST grain. The easier formation of h-phase for thin GST film is indeed a critical issue that should be avoided in manufacturing high density (giga-bit) PCM devices as the sectional size of vertical (dash-typed) phase change film being shrunk to 7.5 nm × 17 nm (ref. 6). Also note that the f-to-h transition becomes much easier when the thickness of GST film sandwiched between ZnS-SiO_2_ cladding films is further scaled from 6 to 2 nm (ref. 34), because the transition temperature is significantly reduced, as well as the energy barrier. Introducing compressive stress to the ultra-thin GST film by encapsulating with suitable dielectric films^34^ or doping with high glass-transition temperature elements^15, 16, 32, 33^ would be helpful for suppressing the formation of the h-phase for high density PCM application.

## Conclusion

In summary, with the aid of *in situ* TEM heating technique, we describe a more comprehensive characterization of the crystallization process of GST thin film. We believe that vacancy ordering phenomenon in f-phase is just a specific fragment of the whole f-to-h transition process. It would be more or less related to the stochastic h-nucleation from f-phase occurred at energetically favorable areas. The abnormal growth of large h-grain at high temperature is mainly driven by surface energy anisotropy. The closest-packed (0001) atomic plane of h-GST grain has the smallest surface energy than those of diversely-oriented f- or h-grains. This driving force would be particularly prominent as the film thickness being greatly diminished and smaller than the average grain size. Thus no matter how complicated the grain boundary would be, the [0001]-oriented h-grain can achieve overall growth. The present results mainly concentrate on the h-grain growth stage, and further study on initially stochastic h-nucleation process will be a great help to more comprehensively understanding the crystallization mechanism of the GST alloy.

## Methods

GST films (~15 nm in thickness) were directly deposited on TEM grids coated with ultra-thin carbon film at room temperature by sputtering GST alloy target. Amorphous SiO_2_ film (~5 nm in thickness), as the anti-oxidation layer, was successively deposited on top of GST films. By using energy-dispersive spectroscopy equipped on the TEM and X-ray fluorescence spectroscopy, the average concentrations of Ge, Sb, and Te elements of the GST films were determined to be 21.4 at.%, 22.6 at.%, and 56.0 at.%. The microstructures of the GST films were characterized by bright-field TEM, SAED, and HRTEM via using JEOL 2100 F TEM under 200 kV. The *in situ* heating crystallization of a-GST film was carried out in a heating holder (Gatan 628) at heating rate of 10 °C/min. During the whole heating process, electron beam was shut off to avoid irradiation effects and only turned on for capturing images^35^.

## Electronic supplementary material

Supplementary Information

Supplementary Movie S1

Supplementary Movie S2

## References

[1] Lam, C. H. Storage Class Memory. *Solid-State and Integrated Circuit Technology* (*ICSICT*), *10th IEEE International Conference on*. 1080–1083 (2010).

[2] Wong H-SP, Salahuddin S (2015). Memory leads the way to better computing. Nat. Nanotech..

[3] Loke D (2012). Breaking the Speed Limits of Phase-Change Memory. Science.

[4] Zhu M (2014). One order of magnitude faster phase change at reduced power in Ti-Sb-Te. Nat. Commun..

[5] Giusca CE (2013). Confined crystals of the smallest phase-change material. Nano Lett..

[6] Kim, I. S. *et al*. High performance PRAM cell scalable to sub-20nm technology with below 4F^2^ cell size, extendable to DRAM applications. *Symp. on VLSI Tech. Dig*. 203–204 (2010).

[7] Cheng, H. Y. *et al*. Novel Fast-switching and High-data Retention Phase-change Memory Based on New Ga-Sb-Ge Material. *IEDM Tech. Dig*. 3.5.1–3.5.4 (2015).

[8] Yamada N (1991). Rapid-phase transitions of GeTe-Sb_2_Te_3_ pseudobinary amorphous thin films for an optical disk memory. J. Appl. Phys..

[9] Friedrich I (2000). Structural transformations of Ge_2_Sb_2_Te_5_ films studied by electrical resistance measurements. J. Appl. Phys..

[10] Yamada N, Matsunaga T (2000). Structure of laser-crystallize Ge_2_Sb_2+x_Te_5_ sputtered thin films for use in optical memory. J. Appl. Phys..

[11] Zhang B (2016). Element-resolved atomic structure imaging of rocksalt Ge_2_Sb_2_Te_5_ phase-change material. Appl. Phys. Lett..

[12] Zhang B (2016). Vacancy structures and melting behavior in rock-salt GeSbTe. Sci. Rep..

[13] Lotnyk A (2016). Real-space imaging of atomic arrangement and vacancy layers ordering in laser crystallised Ge_2_Sb_2_Te_5_ phase change thin films. Acta Materialia.

[14] Matsunaga T (2004). Structures of stable and metastable Ge_2_Sb_2_Te_5_, an intermetallic compound in GeTe-Sb_2_Te_3_ pseudo-binary systems. Acta Crystallogr. Section B..

[15] Cheng, H. Y. *et al*. A High Performance Phase Change Memory with Fast Switching Speed and High Temperature Retention by Engineering the Ge_x_Sb_y_Te_z_ Phase Change Material. *IEDM Tech. Dig*. 3.4.1−3.4.4 (2011).

[16] Zhou X (2014). Understanding Phase-Change Behaviors of Carbon-Doped Ge_2_Sb_2_Te_5_ for Phase-Change Memory Application. ACS Appl. Mater. Interfaces.

[17] Zhang W (2012). Role of vacancies in metal–insulator transitions of crystalline phase-change materials. Nature Mater..

[18] Sun Z (2006). Structure of phase change materials for data storage. Phys. Rev. Lett..

[19] Zhang W (2008). Martensitic transformation in Ge_2_Sb_2_Te_5_ alloy. Adv. Eng. Mater..

[20] Zhang W (2015). How important is the {103} plane of stable Ge_2_Sb_2_Te_5_ for phase-change memory?. Journal of Microscopy.

[21] Park YJ (2006). *In situ* transmission electron microscopy study of the nucleation and grain growth of Ge_2_Sb_2_Te_5_ thin films. Appl. Surf. Sci..

[22] Kim ET (2007). Investigation of the structural transformation behavior of Ge_2_Sb_2_Te_5_ thin films using high resolution electron microscopy. Appl. Phys. Lett..

[23] Song SA (2008). *In situ* dynamic HR-TEM and EELS study on phase transitions of Ge_2_Sb_2_Te_5_ chalcogenides. Ultramicroscopy.

[24] Thompson CV (1998). Grain growth in polycrystalline thin films of semiconductors. Interface Sci..

[25] Chou TC, Nieh TG (1992). Interface-controlled phase transformation and abnormal grain growth of α-Al_2_O_3_ in thin γ-alumina films. Thin Solid Films.

[26] Lankhorst MHR (2005). Low-cost and nanoscale non-volatile memory concept for future silicon chips. Nat. Mater..

[27] Rosenthal T (2011). Real structure and thermoelectric properties of GeTe-rich germanium antimony tellurides. Chem. Mater..

[28] Ross U (2014). Direct imaging of crystal structure and defects in metastable Ge_2_Sb_2_Te_5_ by quantitative aberration-corrected scanning transmission electron microscopy. Appl. Phys. Lett..

[29] Williams, D. B. & Carter, C. B. Transmission Electron Microscopy: A Textbook for Materials Science, 2nd ed. (Springer: New York, 2009).

[30] Mullins WW (1956). Two-dimensional motion of idealized grain boundaries. J. Appl. Phys..

[31] Thompson CV (1985). Secondary grain growth in thin films of semiconductors: theoretical aspects. J. Appl. Phys..

[32] Cheng, H. Y. *et al*. A thermally robust phase change memory by engineering the Ge/N concentration in (Ge, N)_x_Sb_y_Te_z_ phase change material. *IEDM Tech. Dig*. 31.1.1−31.1.4 (2012).

[33] Lee TH (2015). Microscopic mechanism of doping-induced kinetically constrained crystallization in phase change materials. Adv. Mater..

[34] Simpson RE (2010). Toward the ultimate limit of phase change in Ge_2_Sb_2_Te_5_. Nano Lett..

[35] Kooi BJ (2004). *In situ* transmission electron microscopy study of the crystallization of Ge_2_Sb_2_Te_5_. J. Appl. Phys..

