# Supplementary material for: Surface Energy Driven Cubic-to-Hexagonal Grain Growth of Ge2Sb2Te5 Thin Film
Source: Sci Rep. 2017 Jul 19;7:5915. doi: 10.1038/s41598-017-06426-2 (PMC5517630; doi:10.1038/s41598-017-06426-2)
Supplement: Supplementary file 1 — Supplementary Information [file 41598_2017_6426_MOESM1_ESM.doc]

Supplementary Information

**Surface Energy Driven Cubic-to-Hexagonal Grain Growth of Ge_2_Sb_2_Te_5_ Thin Film**

Yonghui Zheng^1^, Yan Cheng^1,†^, Rong Huang^3^, Ruijuan Qi^3^, Feng Rao^1,†^, Keyuan Ding^1,2^, Weijun Yin^1^, Sannian Song^1^, Weili Liu^1^, Zhitang Song^1^, Songlin Feng^1^

^1^State Key Laboratory of Functional Materials for Informatics, Shanghai Institute of Micro-system and Information Technology, Chinese Academy of Sciences, Shanghai 200050, China.

^2^University of the Chinese Academy of Sciences, Beijing 100049, China.

^3^Key Laboratory of Polar Materials and Devices Ministry of Education, East China Normal University, Shanghai 200062, China

^†^Correspondence and requests for materials should be addressed to Y.C. (email: chengyan@mail.sim.ac.cn), or to F.R. (email: fengrao@mail.sim.ac.cn).


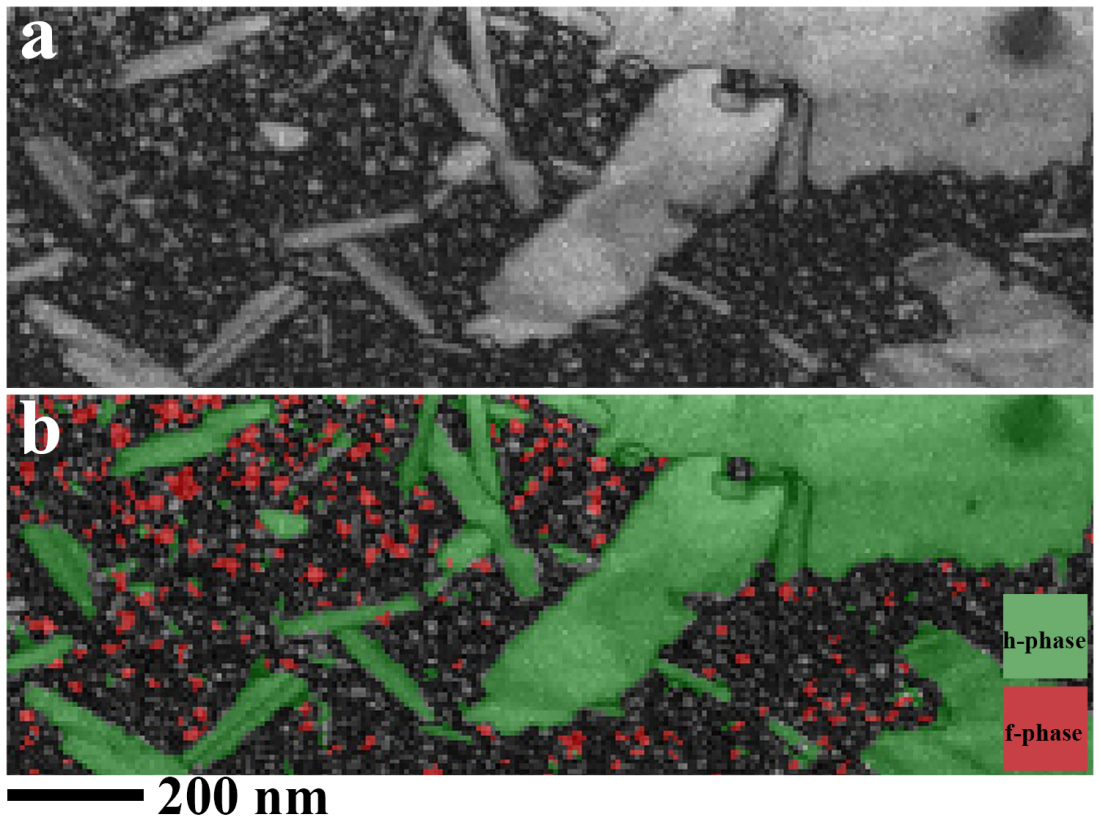


**Supplementary Figure 1.** The snapshot of face-centered cubic (f-) to hexagonal (h-) transitioning moment at 320 ^o^C which is collected by using scanning electron microscope-transmission Kikuchi diffraction (SEM-TKD)^1^ with a step size of 7 nm at 30 kV. (**a**) In the TKD pattern band contrast map, areas with higher-quality diffraction (better crystallinity) present brighter contrast. (**b**) The corresponding electron backscatter diffraction (EBSD) phase map, as compared to Fig.1e in the Main Text, can clearly identify large h-grains (in green) and distinguish scattered small h-grains (also in green) from f-ones (in red). Note that the black areas in both maps represent other unidentified grains with smaller size less than ~7 nm of the scanning step size.

1. Trimby, P. W. Orientation mapping of nanostructured materials using transmission Kikuchi diffraction in the scanning electron microscope. *Ultramicroscopy* **120**, 16–24 (2012).


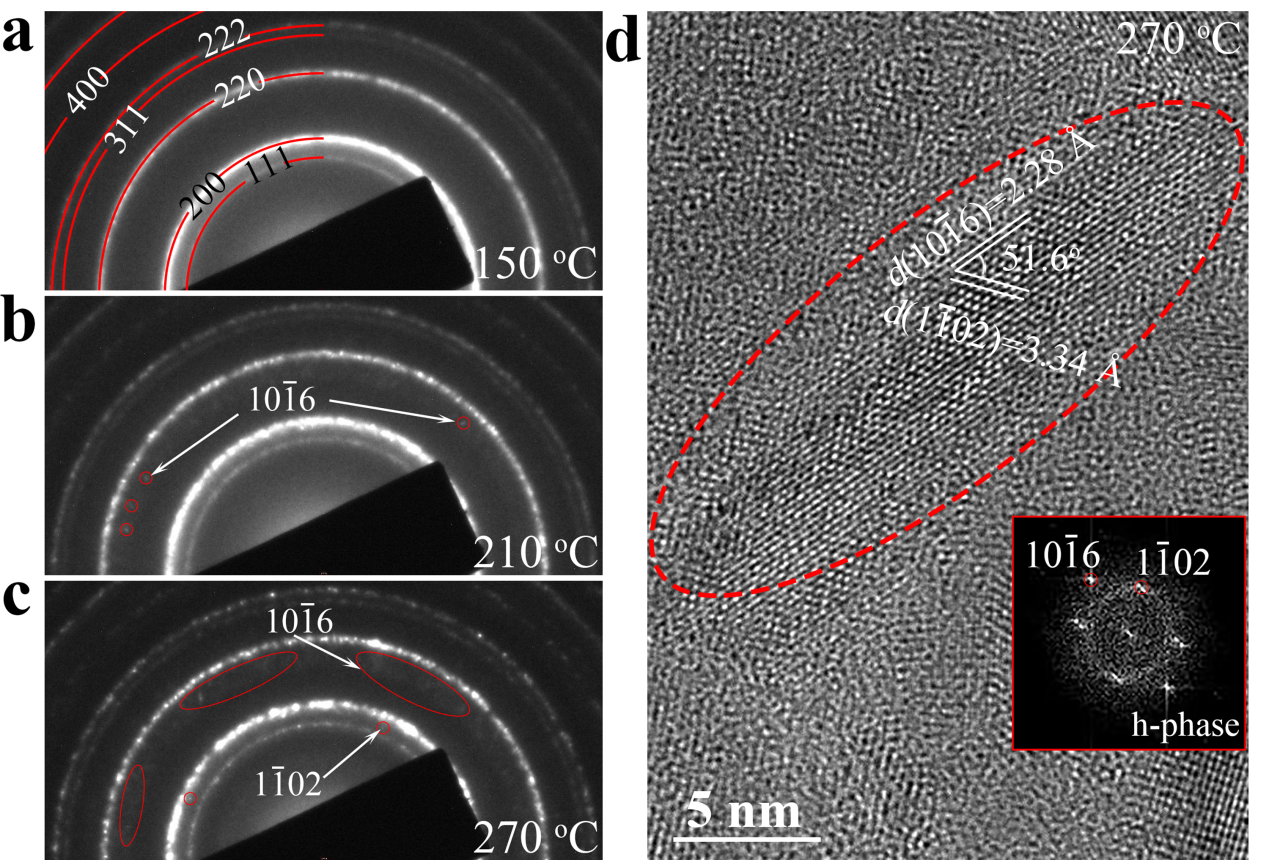


**Supplementary Figure 2.** (**a**) to (**c**) correspond to the zooming selected area electron diffraction (SAED) patterns of Figs. 1b to 1d in the Main Text, respectively. Some extra diffraction spots unfitting with the f-pattern can be identified at 210 ^o^C (b) and 270 ^o^C (c), while they do not appear at 150 ^o^C (a). Note that (
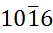
) and (
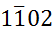
) planes can only co-exist in h-lattice, which can also be identified by the inset fast-Fourier transform (FFT) pattern in (**d**). The advent of these extra diffraction spots demonstrates that some tiny h-grains can be incubated at a pretty low temperature (~210 ^o^C), well below the one (300~350 ^o^C) for large-scale f-to-h grain growth.

**Supplementary** **Movie** **1.** *In situ* transmission electron microscopy movie records the growth process of h-grain which keeps on growing via consuming other small grains. The whole process lasts about 1 min as heating temperature being increased from 320 ^o^C to 330 ^o^C. The observing area is ~2 × 2 μm^2^. The estimated growth rate of the h-grain is about 6.7 nm/s.

**Supplementary** **Movie** **2.** *In situ* high resolution transmission electron microscopy movie records the process for a [0001]-oriented h-grain consuming its neighboring grain at 320 ^o^C. The observing area is ~20 × 20 nm^2^. In this short (~5 s) movie, one can easily observe the swift migration of a h-grain boundary, and there is no rotation for the h-grain and its neighboring grain, indicating the structural transformation during abnormal h-grain growth would mainly related to the atomic rearrangements close to the grain boundary of the dominant h-grain.
